# Aging, stem cells and cancer updated

**DOI:** 10.18632/aging.203535

**Published:** 2021-09-11

**Authors:** Osama A. Elkashty, Arvind Hariharan, Simon D. Tran

**Affiliations:** 1 Craniofacial Tissue Engineering and Stem Cell Laboratory, Faculty of Dentistry, McGill University, Montreal, QC H3A 0C7, Canada

**Keywords:** cancer stem cells, tumorigenesis, epigenetic, head and neck cancer, signaling pathways

Aging is one of the most studied risk factors contributing to malignancies. Studies have shown that there is an increased incidence of malignancies with age; however, it cannot be established that it is the cause, as aging is a natural process [1]. The same biological mechanisms that are responsible for aging, may also be responsible for cancer, which involve epigenetic alterations of cells as well as deterioration of tissues [2]. Often, stem cells are the most targeted for malignant transformation, because of their tendency to accumulate genetic alterations [2]. It is therefore important to gain an in-depth understanding of the mechanisms by which stem cells transform and how aging affect cell propagation. This knowledge is significant for developing added therapeutic options because studying the link between the biological mechanisms of aging and cancer will provide treatment modalities to modify the environmental factors that slow the rate of aging and delay cancer onset.

The hierarchical model suggests that tumorigenesis is attributable to cancer stem cells (CSCs) [3]. CSCs were recently recognized as a third type of stem cell, with a capacity to self–renew and deliver a heterogenous lineage of cancer cells. They are responsible for tumour formation, maintenance, and metastatic growth [2]. Leukemic stem cells were first identified as a sub-population of stem cells with a unique oncogenic potential and was the first recorded evidence that CSCs existed [2]. It is controversial as to how CSCs evolve, and several theories have been proposed in this regard. The most widely accepted theory is that they arise from normal somatic stem cells, but other theories suggest that they could also arise from progenitor or differentiated cells [3]. Tumorigenesis risk occurs due to deregulated cell signalling and changes in the stem cell environment, which contributes to resistance to cell senescence and programmed cell death [2].

The biological processes of aging are still unknown and different theories have been proposed. Aging is often thought to involve the degradation of the stem cell pools, leading to reduced tissue regeneration potential. The propagation of stem cells into CSCs due to genetic and epigenetic alterations is often associated with cancer and could be age-related. Age-dependent DNA damage to stem cells can contribute to tumorigenesis because the stem cells lose the capacity to repair DNA damage [2]. Benign mutations that occur in the stem cell pool at a very early age, and over time, can contribute to the tumorigenesis process [4]. Genome instability can lead to inactivation of tumour suppression genes and activation of oncogenes, allowing them to resist cell apoptosis and senescence. The absence of telomerase leads to shortening of telomeres as the stem cells age and this leads to the downregulation of p53. Due to this, the stem cells generate CSCs with an unstable genome and mutations of tumour suppressor genes and oncogenes [2,4]. According to Feinberg’s model (2005), CSCs and embryonic stem cells have similar epigenetic profiles with similar alterations which explain the rationale behind carcinogenesis pre–dominating in the elderly. This is a significant finding because it can be exploited in future therapeutic modalities [4].

Malignant transformation requires age–associated changes in tissue microenvironment, such as increased inflammation and a decreased immunity [1]. One treatment strategy is through the modification of the risk factors, which will aid in the prevention of malignant transformation of pre-cursor lesions [1]. The normally quiescent CSCs can be targeted early before the onset of cancer, especially in the elderly. Cell signalling pathways such as Wnt, Notch, and Hedgehog signalling pathways are important for the propagation and survival of CSCs. These pathways can interact with each other, causing further resistance to apoptosis of CSCs and self–renewal, which makes it difficult for the identification of treatment strategies [5].

Our laboratory has isolated a subpopulation of CSCs in head and neck cancer (HNC), enriched with cell surface markers CD44 and CD271, which could be used for new therapeutic strategies [6]. The ability of CSCs to evade common chemo-radiotherapeutic regimens also need to be considered. Various genes affected by aging can be targeted to prevent malignant transformation of CSCs. Our laboratory has recently examined the effects of a broccoli extract ingredient (sulforaphane) and its ability to enhance chemotherapy in HNC, by targeting the associated CSCs isolated previously [7]. Our findings suggest that the broccoli extract was able to increase the cytotoxicity of chemotherapeutic agents and inhibited the formation of CSCs [7,8]. We also found that the possible mechanism of action was through the stimulation of the apoptotic pathway and the targeting of genes like SOX2 and OCT4, both indicators of stemness [8].

In summary, even though aging is a natural process, it can be considered as a risk factor for malignancy. Further research is required to determine the reasons behind the increased incidence of cancer in the aging population. The transformation of adult stem cells into CSCs is a possible factor that cannot be overlooked. CSCs are often the drivers of resistance to conventional therapeutic regimens, and it is important to consider newer treatments to target them to prevent metastasis and the malignant transformation of pre-cursor lesions. The findings in our laboratory have indeed opened avenues for the identification of these cells and upgrading current treatment strategies. Further research in studying the mechanisms between aging and CSC formation is pivotal for the translation of new treatments into human use.
